# Tuberculosis Presenting as Ruptured Liver Abscess and Inferior Vena Cava Thrombosis in a Pediatric Patient

**DOI:** 10.31486/toj.23.0030

**Published:** 2023

**Authors:** Nadia Nazir Jatoi, Marium Ashraf, Varisha Fatima Shaikh, Sharmeen Nasir, Hafsa Nazir Jatoi

**Affiliations:** ^1^ Department of Pediatrics, Dow University of Health Sciences, Dr. Ruth K. M. Pfau Civil Hospital, Karachi, Pakistan; ^2^ Department of Internal Medicine, Dow Medical College, Dow University of Health Sciences, Karachi, Pakistan

**Keywords:** *Antitubercular agents*, *liver abscess*, Mycobacterium tuberculosis, *thrombosis*, *tuberculosis*, *vena cava–inferior*

## Abstract

**Background:** Tuberculosis is a leading cause of mortality and morbidity in many countries across the world, including Pakistan. While systemic tuberculosis can involve any organ of the body, tubercular liver abscess is a rare presentation.

**Case Report:** We report the case of an 8-year-old female from a developing country where tuberculosis poses a significant burden on the health care system. The patient presented with fever and weight loss for 6 months and abdominal pain for 14 days. On examination, she had tenderness and guarding over the right hypochondrium. Investigations revealed neutrophilic predominance in the complete blood count and elevated C-reactive protein. Imaging of the abdomen revealed ruptured liver abscess, extensive abdominal lymphadenopathy, and thrombus in the inferior vena cava. Gastric secretions were positive for *Mycobacterium tuberculosis*. Treatment included antitubercular and antithrombotic therapy. The patient was closely followed until she had completed the 1-year course of antitubercular therapy and was symptom-free.

**Conclusion:** In tuberculosis-endemic countries, physicians should keep a high index of suspicion for tuberculosis in children who present with liver abscess and multisystem involvement.

## INTRODUCTION

Among countries with a high burden of tuberculosis, Pakistan ranks fifth. The prevalence, incidence, and mortality per 100,000 population per year in Pakistan are 348, 276, and 34, respectively.^1^ Tuberculosis can affect all organs in the human body.^2^ While hepatic tuberculosis is a common condition, tubercular liver abscess is relatively uncommon, even in countries where tuberculosis is a major public health concern,^3^ and is commonly misdiagnosed as a liver abscess of another etiology.^2^ The infrequent association of inferior vena cava thrombosis in tuberculosis has also been reported.^4^ We describe the case of an 8-year-old female who presented with a ruptured liver abscess and inferior vena cava thrombus that proved to be a case of disseminated tuberculosis.

## CASE REPORT

An 8-year-old female who had not been vaccinated for any infectious diseases including tuberculosis presented to the pediatric department at a tertiary care hospital in Karachi, Pakistan, with complaints of fever for 6 months, as well as abdominal pain and generalized edema for the prior 14 days. The fever had been intermittent and low grade and was associated with progressive weight loss. During the prior 14 days, the patient's fever became high grade, was documented up to 102 °F, and was associated with abdominal pain and distention. Pain was localized to the right hypochondrium and epigastrium regions, was severe in intensity, and was associated with reduced appetite. According to the patient, the edema started peripherally in the lower extremities and increased progressively to involve the legs and then the abdomen. She also complained of occasional headaches.

On examination, she was a cachectic, pale, conscious, and oriented child, weighing 18 kg. She was febrile, with tachycardia and tachypnea on presentation. Bilateral peripheral pitting edema was noted. Her abdomen was distended and tense on examination with severe tenderness and guarding in the right hypochondrium. Visceral palpation and percussion were not possible because of severe pain.

The patient was admitted and administered intravenous (IV) paracetamol 10 mg/kg every 8 hours and IV fluid 0.9% dextrose saline 1,400 mL over 24 hours. Complete blood count revealed microcytic hypochromic anemia with hemoglobin 7.0 g/dL (reference range, 11.5-13.5 g/dL), white blood cells 13,760/mm^3^ (reference range, 4,000-11,000/mm^3^), neutrophil count 84% (reference range, 33%-55%), and platelet count 276,000/mm^3^ (reference range, 15,000-350,000/mm^3^). Erythrocyte sedimentation rate was 20 mm/hr (reference, <10 mm/hr) at the end of the first hour, and C-reactive protein was 136 mg/dL (reference, <5 mg/dL). Blood urea nitrogen, creatinine, and liver function tests were unremarkable. Serum electrolytes showed hypokalemia with a potassium level of 2.5 mEq/L (reference range, 3.5-5.0 mEq/L); other electrolytes were normal.

**Figure 1.**
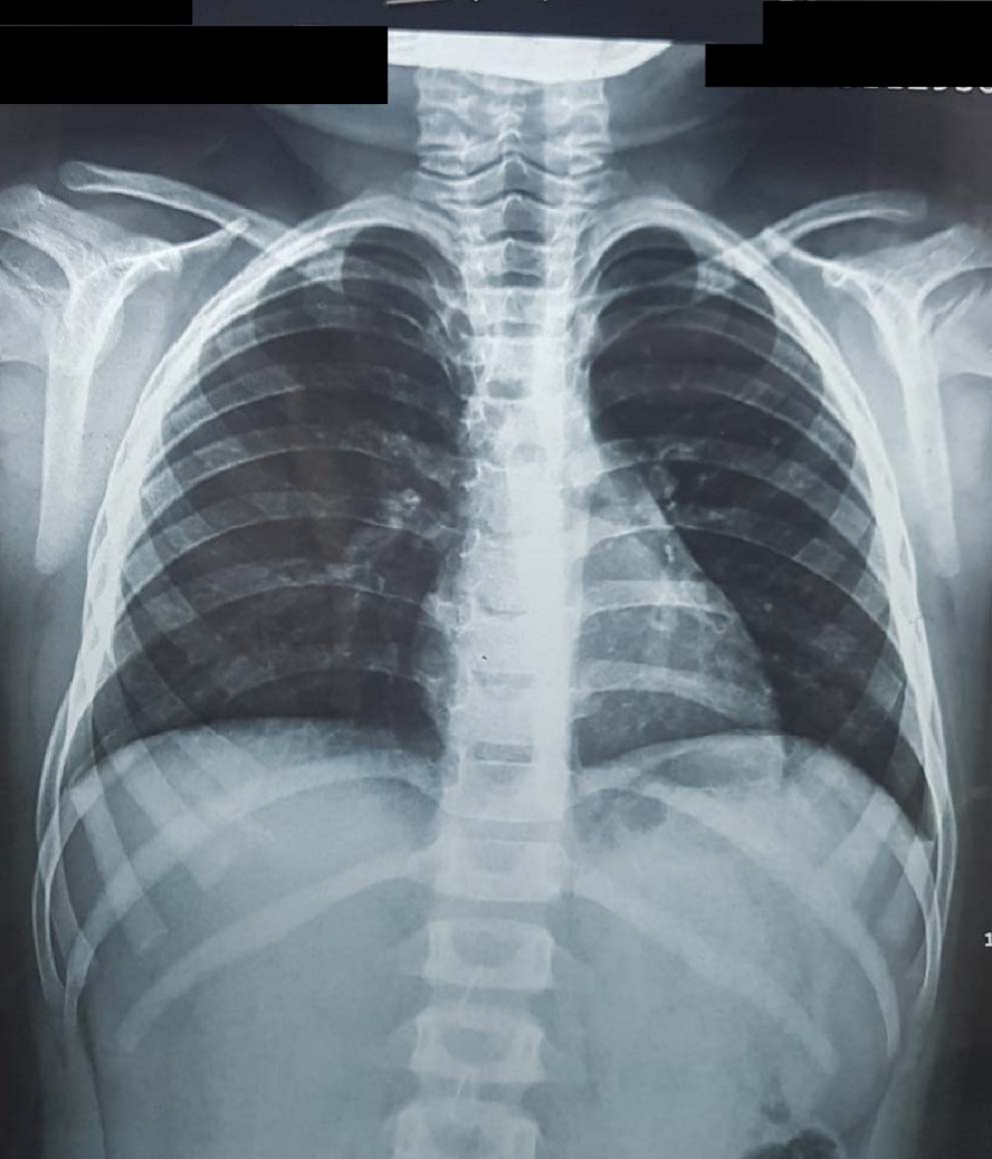
Chest x-ray was unremarkable, showing no signs of tuberculosis.

Chest x-ray was unremarkable (Figure 1). Ultrasound of the abdomen showed a normal-sized liver with smooth margins, altered echotexture, and a well-defined hypoechoic lesion in the right lobe measuring 1.8 × 2.4 cm with perihepatic collection. Findings were suggestive of ruptured liver abscess with minimal ascites and fluid-filled bowel loops with sluggish movement. Color Doppler computed tomography (CT) scan showed no flow, suggestive of a ruptured abscess. No pleural effusion was seen, and the rest of the abdominal viscera appeared normal with no free fluid. Stool routine and microscopic investigations showed no cyst or ova; amoebic serology of the blood was negative. HIV testing to rule out secondary acquired immunodeficiency was negative. As the child was 8 years of age and had no history of recurrent infections, workup for primary immunodeficiency was not warranted.

Because the abscess was already ruptured, it was not possible to aspirate pus for culture. Broad-spectrum antibiotics were started to treat bacterial and amoebic liver abscess, as per institutional protocol. The patient received IV metronidazole 10 mg/kg every 8 hours and IV cefotaxime 150 mg/kg/day in a divided dose every 8 hours.

**Figure 2.**
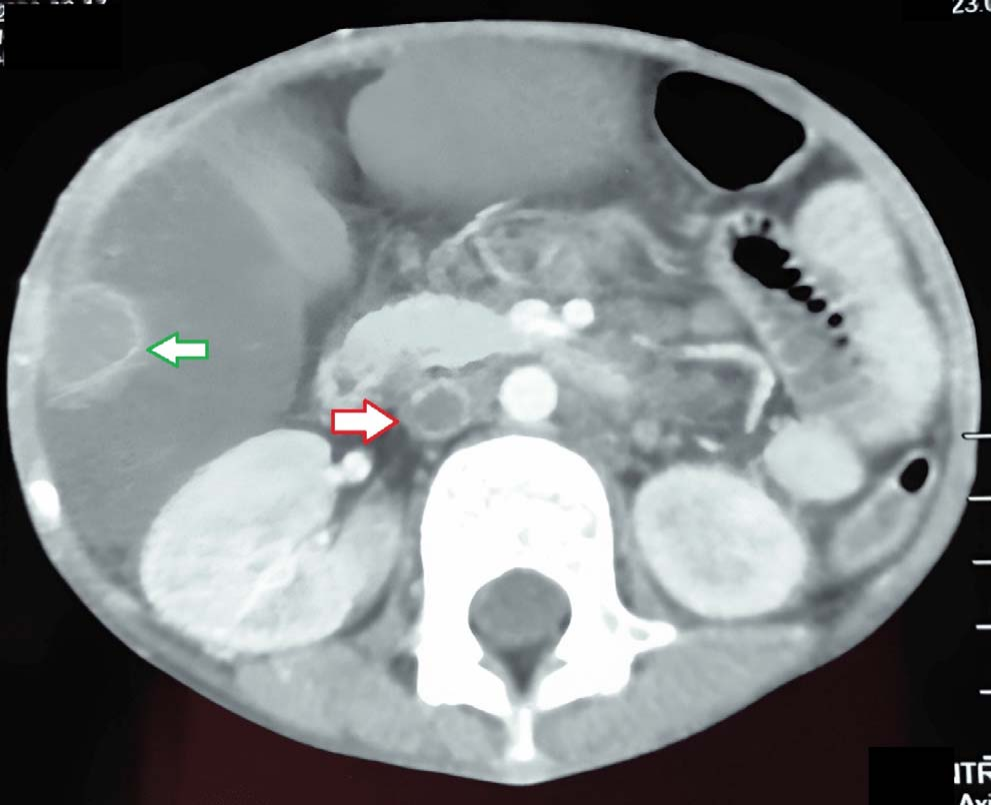
Contrast-enhanced computed tomography of the abdomen showed thrombus of the inferior vena cava starting at the level of the renal vein and extending toward the common iliac and external iliac vein on the right side (red arrow) and a hypoechoic mass in the liver (green arrow).

Contrast-enhanced CT of the abdomen revealed hepat-ic capsule thickening and peritoneal thickening along the liver with subcapsular and perihepatic collection. Extensive mesenteric and para-aortic lymphadenopathy was seen, as well as thrombosis of the inferior vena cava starting at the level of the renal vein and extending toward the common iliac and external iliac vein on the right side (Figure 2). For thrombosis, subcutaneous low molecular weight heparin (LMWH) 20 mg twice daily was given for 2 weeks followed by tablet warfarin 5 mg once daily, with serial international normalized ratio (INR) monitoring to maintain an INR between 2.0 and 3.0.

**Figure 3.**
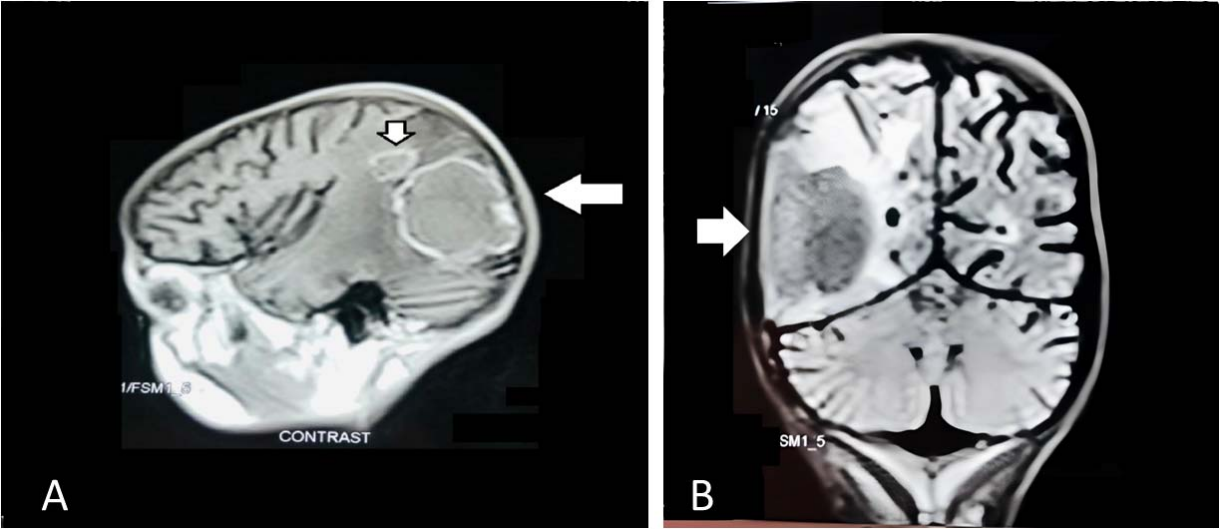
Magnetic resonance imaging of the brain in (A) sagittal and (B) coronal view showed a large ring-enhancing lesion (4.2 × 3.6 cm) in the right posterior temporal and parietal region with marked surrounding edema (horizontal arrow in A and B), as well as a small ring-enhancing lesion (1.3 × 1.4 cm) adjacent to the primary lesion (vertical arrow in A).

On the fifth day of admission, the patient had 3 episodes of generalized tonic-clonic seizures. Magnetic resonance imaging (MRI) of the brain showed a large ring-enhancing lesion (4.2 × 3.6 cm) in the right posterior temporal and parietal region with marked surrounding edema. A small ring-enhancing lesion (1.3 × 1.4 cm) was adjacent to the primary lesion (Figure 3). Cerebrospinal fluid detailed report was normal, and culture was negative. For seizures, the patient was initially started on IV levetiracetam 10 mg/kg every 8 hours; after 48 hours, she was switched to the oral form at the same dose.

Polymerase chain reaction (PCR) analysis of gastric secretions for detection of *Mycobacterium tuberculosis* was positive. Mantoux tuberculin skin test was also positive, with an induration of 12 mm.

The patient was started on antitubercular therapy with a 4-drug regimen: isoniazid 180 mg (10 mg/kg), pyrazinamide 540 mg (30 mg/kg), rifampicin 270 mg (15 mg/kg), and ethambutol 360 mg (20 mg/kg), all once daily, along with tablet pyridoxine 50 mg once daily.

Follow-up ultrasound of the abdomen after 2 weeks of antitubercular therapy and antithrombotic therapy showed marked regression in the abscess size.

The patient's hospital stay was 3 weeks. She was discharged with stable vitals and advised to continue the 4-drug antitubercular regimen (isoniazid, rifampicin, pyrazinamide, and ethambutol) for a total of 2 months, followed by a 2-drug regimen (isoniazid and rifampicin) for 10 more months in the same doses as administered in the hospital. Tablet pyridoxine 50 mg was to be taken daily. Tablet levetiracetam 10 mg/kg and warfarin 5 mg once daily were also prescribed. The parents were counseled to keep the child compliant with the medication and to maintain her overall hygiene. The patient was asked to follow up at 2-week intervals for 2 months and then monthly thereafter for 1 year for clinical symptoms monitoring, imaging (ultrasound) to check for the dissolution of thrombus, and INR monitoring.

After 2 weeks, the patient was back in school and compliant with the antitubercular therapy. Serial ultrasounds showed that the thrombus was resolving. CT of the abdomen at 2-month follow-up showed no signs of thrombus, so warfarin was discontinued. Brain CT scan after 6 months showed that the lesions in the brain were completely resolved, so levetiracetam tapering was started. The patient was closely followed until she had completed the 1-year course of antitubercular therapy and was symptom-free.

## DISCUSSION

The first case of tubercular liver abscess was reported in 1945,^5^ and cases have continued to be reported to the present (2021).^3,6-10^ While hepatic tuberculosis is expected in disseminated tuberculosis, isolated hepatic tuberculosis has an incidence <1%, and tubercular liver abscess is found in only 0.3% of patients with hepatic tuberculosis.^11^ Rai et al reported a case of tubercular liver abscess in a 70-year-old diabetic male who had no history or findings of tuberculosis in the lungs or abdomen.^6^ Although tubercular liver abscess is generally secondary to lung or gastrointestinal tract tuberculosis,^10^ the chest x-ray can be normal in isolated tubercular liver abscess, suggesting that isolated tubercular liver abscess should be kept in the differentials of bacterial or amoebic liver abscess in countries where tuberculosis is prevalent. Cases of tubercular liver abscess have been reported in immunocompetent patients,^3,7,9^ in the pediatric population,^8^ and in older age groups.^6,10^

The clinical presentation of the disease usually involves fever, weight loss, right upper quadrant pain, anorexia, and hepatomegaly, with jaundice being a rare finding.^6,7^ Complete blood count shows anemia and leukocytosis, while liver function tests are usually normal.^9^ Ultrasound shows a hypoechoic mass^10^ and can also help visualize the number of abscesses. Patients may have more than 1 liver abscess.^9^ CT can be used for assessment and shows hypoechoic nodular lesions with different stages of the disease such as granulomatous tubercles with or without caseous necrosis to fibrosis and calcifications in different healing stages. Contrast enhancement can only be seen in peripheral lesions.^10^ Conclusive diagnosis is made by microbiological assessment of positive finding of the acid-fast bacilli in pus aspirate on biopsy.^9^ Culture is the gold standard; however, the incubation period can delay the results. PCR can be used when rapid diagnosis of *M tuberculosis* is necessary.^7,9^ DNA amplification by PCR is the most common technique, with a specificity of 98% and sensitivity of 92.4%.^12^

Tuberculosis can also complicate cases with inferior vena cava thrombosis as noted in our patient. Underlying hemophilic conditions might increase the chances of thrombosis in a patient with tuberculosis. However, matted tuberculous lymph nodes can also cause inferior vena cava thrombosis.^13^ Because inferior vena cava thrombosis is associated with a major risk for pulmonary embolism, prompt diagnosis and treatment are crucial. Diagnosis of inferior vena cava thrombosis usually requires CT or MRI.^13^

Treatment should commence immediately after the diagnosis has been made. The treatment options for tubercular liver abscess are antitubercular therapy of at least 6 months^9^ to 1 year.^3^ According to some studies, percutaneous drainage can also be beneficial.^14^ Kubota et al used transcatheter infusion of antitubercular drugs with successful results.^15^ Surgery is confined to patients who do not improve with medical therapy and those with large and multiple abscesses.^14^ Treatment for thrombosis is LMWH.^13^

Our patient presented with tuberculomas that manifested as seizures. Tuberculous brain abscesses are a common presentation of tuberculosis and present with symptoms of confusion, coma, seizures, multiple cranial nerve deficits, focal neurologic signs, and stroke, along with vomiting, focal and generalized convulsions, and irritability. Among the 25% to 30% of the world population infected with *M tuberculosis*, central nervous system tuberculosis is reported in 1% to 2% of individuals diagnosed with active tuberculosis.^16^ Seventy percent to 80% of central nervous system tuberculosis cases are tubercular meningitis.^16^

Tuberculomas are especially prevalent in India and Asia; of all the intracranial space–occupying lesions, 20% to 30% are tuberculomas, and despite the currently available measures for diagnosis and treatment, central nervous system tuberculosis has a high mortality risk of 15% to 40%.^16^

Scarce literature is available about complications if a tubercular liver abscess is left untreated; however, Patel et al reported a 14-year-old with neurologic manifestations, signifying tuberculous meningitis can be a direct complication of primary tubercular liver abscess.^17^

## CONCLUSION

Tuberculosis, a preventable and treatable disease with high morbidity in developing countries, can present with widespread and severe involvement of various organs and rare presentations such as liver abscess and intravenous thrombosis. Our case highlights the need for a high index of suspicion in patients from tuberculosis-endemic areas who present with multisystem, widespread involvement or even isolated infections.

## Notes

*This article meets the Accreditation Council for Graduate Medical Education and the American Board of Medical Specialties Maintenance of Certification competencies for Patient Care and Medical Knowledge.*

## References

[1] About Us. National TB Control Programme – Pakistan. Accessed August 2, 2023. ntp.gov.pk/about-us/

[2] Dey J, Gautam H, Venugopal S, Tuberculosis as an etiological factor in liver abscess in adults. Tuberc Res Treat. 2016;2016:8479456. doi: 10.1155/2016/8479456 27595021 PMC4995316

[3] Baveja C, Gumma V, Chaudhary M, Jha H. Primary tubercular liver abscess in an immunocompetent adult: a case report. J Med Case Rep. 2009;3:78. doi: 10.1186/1752-1947-3-78 19946554 PMC2783077

[4] Raj M, Agrawal A. Inferior vena cava thrombosis complicating tuberculosis. N Z Med J. 2006;119(1244):U2279. 17072354

[5] Ruppanner E. Metastatische infektion eines tuberkulösen senkungsabszesses mit dem *Bacillus funduliformis* [Metastatic infection of a tubercular subsidence abscess with the *Bacillus funduliformis*]. Schweiz Med Wochenschr. 1945;75:1089. 21010421

[6] Rai R, Tripathi VD, Rangare V, Reddy DS, Patel P. Isolated tubercular liver abscess in an elderly diabetic successfully treated with systemic antitubercular drugs. J Pak Med Assoc. 2012;62(2):170-172. 22755384

[7] Shokouhi S, Toolabi K, Tehrani S, Hemmatian M. Tuberculous liver abscess in an immunocompetent patient: a case report. Tanaffos. 2014;13(3):49-51. 25713592 PMC4338053

[8] Kashyap B, Gupta N, Singha K, Clinico-immunological profile in a case of hepatic tubercular abscess: a rare manifestation of extrapulmonary TB in an immunocompetent child. Indian J Tuberc. 2021;68(1):160-162. doi: 10.1016/j.ijtb.2020.09.001 33641843

[9] Devi S, Mishra P, Sethy M, Thakur GS. Isolated tubercular liver abscess in a non-immunodeficient patient: a rare case report. Cureus. 2019;11(12):e6282. doi: 10.7759/cureus.6282 31911874 PMC6939970

[10] Hayashi M, Yamawaki I, Okajima K, Tomimatsu M, Ohkawa S. Tuberculous liver abscess not associated with lung involvement. Intern Med. 2004;43(6):521-523. doi: 10.2169/internalmedicine.43.521 15283192

[11] Peters NJ, Samujh R, Gunasekaran V, Sodhi KS, Duseja R. Pediatric pseudotumoral hepatic tuberculosis. A great mimicker!! J Indian Assoc Pediatr Surg. 2022;27(3):368-370. 35733599 10.4103/jiaps.JIAPS_58_21 PMC9208692

[12] Zakham F, Lahlou O, Akrim M, Comparison of a DNA based PCR approach with conventional methods for the detection of *Mycobacterium tuberculosis* in Morocco. Mediterr J Hematol Infect Dis. 2012;4(1):e2012049. doi: 10.4084/MJHID.2012.049 22973493 PMC3435128

[13] Abid R, Oueslati I, Bousetta N, Inferior vena cava thrombosis complicating tuberculosis. Ann Clin Case Rep*.* 2018;3(1):1534.

[14] Chen HC, Chao YC, Shyu RY, Hsieh TY. Isolated tuberculous liver abscesses with multiple hyperechoic masses on ultrasound: a case report and review of the literature. Liver Int. 2003;23(5):346-350. doi: 10.1034/j.1478-3231.2003.00861.x 14708895

[15] Kubota H, Ageta M, Kubo H, Wada S, Nagamachi S, Yamanaka T. Tuberculous liver abscess treated by percutaneous infusion of antituberculous agents. Intern Med. 1994;33(6):351-356. doi: 10.2169/internalmedicine.33.351 7919622

[16] Gupta M, Munakomi S. CNS tuberculosis. In: *StatPearls*. Treasure Island (FL): StatPearls Publishing; February 12, 2023. 36256788

[17] Patel R, Choksi D, Poddar P, Shah K, Ingle M, Sawant P. Primary tubercular liver abscess complicated by tubercular meningitis in portal cavernoma cholangiopathy. ACG Case Rep J. 2016;3(4):e196. doi: 10.14309/crj.2016.169 28119947 PMC5226201

